# A Modified Tumor-Node-Metastasis Classification for Stage III Colorectal Cancers Based on Treating Tumor Deposits as Positive Lymph Nodes

**DOI:** 10.3389/fmed.2020.571154

**Published:** 2020-10-15

**Authors:** Jun-Peng Pei, Chun-Dong Zhang, Xiang Fu, Yong Ba, Shuai Yue, Zhe-Ming Zhao, Dong-Qiu Dai

**Affiliations:** ^1^Department of Gastrointestinal Surgery, The Fourth Affiliated Hospital of China Medical University, Shenyang, China; ^2^Department of Gastrointestinal Surgery, Graduate School of Medicine, University of Tokyo, Tokyo, Japan; ^3^Cancer Center, The Fourth Affiliated Hospital of China Medical University, Shenyang, China

**Keywords:** colorectal cancer, tumor deposit, prognosis, lymph node, overall survival

## Abstract

**Background:** The tumor-node-metastasis classification of the American Joint Committee on Cancer classified tumor deposits (TDs) in patients with colorectal cancer (CRC) without lymph node (LN) metastasis as N1c, but the classification of TDs in patients with LN metastases remains controversial. This study investigated the probability of regarding TDs as positive LNs (pLNs) in pN stage and estimated its prognostic ability in CRC.

**Methods:** We used the Surveillance, Epidemiology, and End Results program to analyze CRC patients who underwent surgical therapy (14,906 training cohort, 6,384 validation cohort). A modified pN stage (mpN) was identified using the number of pLNs plus TDs. Overall survival (OS) was analyzed using the Kaplan–Meier survival curves, and significant prognostic factors were identified by univariate and multivariate analyses. Prognostic ability was estimated using the area under the curve (AUC), calibration curve, and the Akaike's information criterion (AIC). Clinical benefit was measured by the decision curve analyses (DCA). The results were validated using the validation cohort.

**Results:** Both the pN and mpN stages were independent prognostic factors in CRC according to univariate and multivariate analyses. The AUC analysis showed that the mpN stage had better prognostic discrimination for OS than the pN stage (0.612 vs. 0.605, *P* < 0.001). The AIC demonstrated that the mpN stage also showed superior model-fitting compared with the pN stage (49,756 vs. 49,841). The DCA further revealed that the mpN stage had better clinical benefits than the pN stage. The validation cohort showed similar findings.

**Conclusions:** We concluded that counting TDs as pLNs may be superior to the pN stage when assessing the prognosis of CRC patients.

## Introduction

Colorectal cancer (CRC) is a common malignant tumor with high mortality (1). The American Joint Committee on Cancer (AJCC) tumor-node-metastasis (TNM) classification is one of the most important standards for risk assessment (2) and has been revised several times, particularly in relation to the pN stage (3–6). The TNM classification is the most basic and common classification for evaluating post-operative patient prognosis.

Tumor deposits (TDs) are defined as focal aggregates of cancer cells in the mesentery, subserosa, or pericolic tissues (7). Recent studies identified TDs as an important prognostic factor for overall survival (OS) in patients with CRC (8–11), with patients with TDs having a poorer prognosis than those without TDs. TDs were first included in the 7th AJCC TNM classification, and the pN1c stage was proposed, and pN0 CRCs with TD involvement have been reclassified into pN1c as a novel substage (5). The latest AJCC 8th TNM classification remains unchanged in this regard (6). However, although the 8th AJCC TNM classification of CRC suggests that the number of TDs should be recorded, there is no recommendation on how to categorize pN+ patients with TDs, which might influence the accuracy of CRC staging.

The possibility of counting TDs as the number of positive lymph nodes (pLNs) in the CRC has recently been investigated, and the results indicated that counting TDs as pLNs improved the predictive ability for assessing prognosis and survival in patients with CRC (12, 13). However, previous studies only included a limited number of Asian patients, with no validation cohorts from western countries. The demographic and pathological characteristics of CRC patients in Asian countries may be different from those in Western countries; therefore, it is necessary to further investigate it by Western populations. On the other hand, in the latest AJCC 8th TNM classification of gastric cancer, pathologic evaluation of LNs requires their removal and histologic examination to assess the total numbers of LNs and TDs without evidence of remnant LN tissue that were counted as pLNs (6). The current study therefore investigated the use of counting TDs as pLNs in the TNM classification, and confirmed the prognostic value of this approach in patients with CRC.

## Materials and Methods

### Patients

Using the Surveillance, Epidemiology, and End Results (SEER) program, 992,325 CRC patients were screened between 1975 and 2016 (14). The inclusion criteria were as follows: (1) CRCs; (2) informative variables; (3) aged between 18 and 75 years; (4) primary and single tumor; (5) no distant metastasis (M0); (6) patients with LN metastasis (pN+); (7) received surgical treatment; (8) no pre-operative therapy; and (9) longer than 1-month survival. Exclusion criteria were as follows: (1) lacking available information; (2) aged <18 or >75 years; (3) multiple cancers; (4) with distant metastasis (M1); (5) patients who were categorized as pN1c or had no pLNs; (6) no surgical treatment; (7) with pre-operative therapy; and (8) less than 1-month post-operative survival. Finally, a total of 21,290 stage III CRCs were included and randomized into a training (*n* = 14,906) and validation cohorts (*n* = 6,384), with a randomized ratio of 7:3.

### Categorization

**Figure 1:**
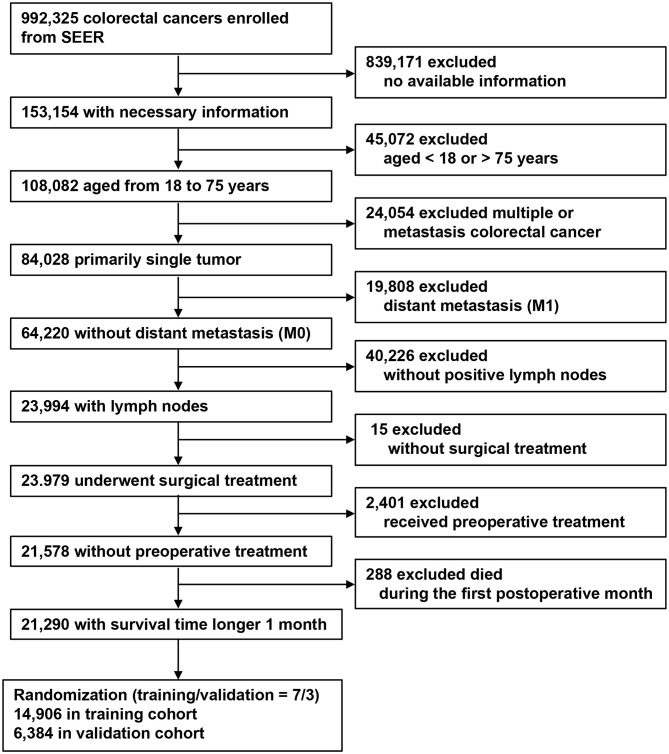
Flow chart for patient selection and study development.

The patient selection process is shown in Figure 1. All patients were categorized according to the AJCC 8th TNM classification. We counted TDs as pLNs in the modified pN stage. The modified pN classification and modified TNM classifications were recorded as mpN stage and mTNM classification, respectively.

pN stage is defined as the regional lymph nodes involvement. The details of pN stage are as follows: pN1a: 1 pLN; pN1b: 2–3 pLNs; pN2a: 4–6 pLNs; pN2b: ≥7 pLNs. The TNM staging system of stage III CRC is as follows: stages IIIA (T1N1, T2N1, and T1N2a), IIIB (T3N1, T4aN1, T2N2a, T3N2a, T1N2b, T2N2b), IIIC (T4aN2a, T3N2b, T4aN2b, T4bN1, and T4bN2) (6).

The details of mpN stage were as follows: mpN1a, 1 pLN, or TD; mpN1b, 2–3 pLNs plus TDs; mpN2a: 4–6 pLNs plus TDs; and mpN2b: ≥7 pLNs plus TDs. The mTNM staging system of stage III CRC was accordingly as follows: stages IIIA (T1mpN1, T2mpN1, and T1mpN2a), IIIB (T3mpN1, T4ampN1, T2mpN2a, T3mpN2a, T1mpN2b, and T2mpN2b), and IIIC (T4ampN2a, T3mpN2b, T4ampN2b, T4bmpN1, and T4bmpN2).

### Statistical Analyses

Continuous variables were presented as medians or means with standard deviation. Survival curves were created using Kaplan–Meier methods with log-rank tests. Multivariate analyses were conducted using a Cox proportional hazards model. The predictive discrimination abilities of the models were assessed using the area under the receiver-operating characteristic curves (AUCs), and the AUCs were compared using the Hanley and McNeil tests. Model-fitting performances were assessed using the Akaike's information criterion (AIC) (15). Higher AUC values demonstrated superior predictive discrimination, and lower AIC values demonstrated better model-fitting performances. Clinical benefits were estimated by decision curve analyses (DCAs) (16, 17).

All data were analyzed using SPSS 22.0 statistical package (SPSS Inc., Chicago, IL, USA), MedCalc (Version 15.2, Ostend, Belgium), and R version 3.5.6 (http://www.r-project.org/). All tests were two-sided, and *P*-values less than 0.05 were considered statistically significant. A data use agreement with SEER has been obtained. The approval of the institutional review board was not required as the SEER database holds publicly available de-identified data.

## Results

### Patient Characteristics

The baseline characteristics of stage III CRC patients are shown in Table 1. The training cohort included 1,949 (13.1%) patients with TDs and 12,957 (86.9%) patients without TDs. Among these, 12,128 (81.4%) patients had colon cancers and 2,778 (18.6%) had rectal cancers. There were no significant differences in the distributions of baseline characteristics between the training and validation cohorts.

**Table 1:** Baseline characteristics and univariate analysis of training and validation cohorts.

**Variables**	**Training cohort^a^**			**Validation cohort^a^**		
	**No. of patients (%)**	**5-Y OS (%)**	***P-* value**	**No. of patients (%)**	**5-Y OS (%)**	***P*-value**
Location			0.001			0.045
Colon	12,128 (81.4)	70.8		5,135 (80.4)	71.9	
Rectum	2,778 (18.6)	74.5		1,249 (19.6)	75.4	
Sex			0.001			0.002
Female	6,990 (46.9)	73.4		3,071 (48.1)	75.1	
Male	7,916 (53.1)	69.8		3,313 (51.9)	70.2	
Race			<0.001			<0.001
White	11,004 (73.8)	72.3		4,757 (74.5)	73.1	
Black	2,120 (14.2)	65.2		877 (13.7)	66.2	
Other	1,690 (11.3)	72.8		712 (11.2)	75.4	
Unknown	92 (0.6)	96.6		38 (0.6)	97.4	
Age, year			<0.001			<0.001
≤ 60	7,676 (51.5)	76.4		3,296 (51.6)	76.7	
>60	7,230 (48.5)	66.4		3,088 (48.4)	68.1	
Size, cm			<0.001			<0.001
≤ 4.5	7,934 (53.2)	74.2		3,344 (52.4)	75.6	
>4.5	6,479 (43.5)	67.8		2,835 (44.4)	68.8	
Unknown	493 (3.3)	76.1		205 (3.2)	73.1	
Histological grade			<0.001			<0.001
Grade I	783 (5.3)	73.7		327 (5.1)	78.7	
Grade II	10,509 (70.5)	74.4		4,499 (70.5)	75.6	
Grade III	2,784 (18.7)	63.4		1,214 (19)	63.5	
Grade IV	597 (4.0)	57.4		242 (3.8)	58.0	
Unknown	233 (1.6)	66.8		102 (1.6)	62.8	
T stage			<0.001			<0.001
T1	919 (6.2)	86.9		405 (6.3)	86.8	
T2	1,664 (11.2)	86.7		712 (11.2)	87.3	
T3	9,494 (63.7)	72.7		4,047 (63.4)	73.9	
T4a	2,023 (13.6)	55.4		843 (13.2)	53.6	
T4b	806 (5.4)	47.1		377 (5.9)	54.1	
N stage			<0.001			<0.001
pN1a	5,113 (34.3)	79.5		2,248 (35.2)	81.6	
pN1b	4,775 (32.0)	74.1		2,013 (31.5)	74.5	
pN2a	2,884 (19.3)	67.3		1,255 (19.7)	66.4	
pN2b	2,134 (14.3)	52.6		868 (13.6)	53.7	
mpN stage			<0.001			<0.001
mpN1a	4,669 (31.3)	80.5		2,052 (32.1)	83.1	
mpN1b	4,702 (31.5)	75.2		1,985 (31.1)	75.1	
mpN2a	3,010 (20.2)	68.0		1,291 (20.2)	67.4	
mpN2b	2,525 (16.9)	51.9		1,056 (16.5)	53.0	
Tumor-node-metastasis (TNM) staging system			<0.001			<0.001
IIIA	2,277 (15.3)	87.4		983 (15.4)	87.4	
IIIB	9,473 (63.6)	74.1		4,073 (63.8)	74.7	
IIIC	3,156 (21.2)	52.2		1,328 (20.8)	54.6	
mTNM staging system			<0.001			<0.001
IIIA	2,247 (15.1)	87.7		964 (15.1)	88.0	
IIIB	9,144 (61.3)	74.9		3,929 (61.5)	75.6	
IIIC	3,515 (23.6)	51.7		1,491 (23.4)	53.9	
TD status			<0.001			<0.001
Negative	12,957 (86.9)	73.5		5,550 (86.9)	74.5	
Positive	1,949 (13.1)	54.8		834 (13.1)	56.4	

*mpN, modified pN; mTNM, modified TNM; No., number; OS, overall survival; TD, tumor deposit; 5-Y, 5-year*.

a *Ratio of training and validation cohorts is 7:3 by randomized number using R software*.

### Univariate and Multivariate Analyses

Univariate analysis identified tumor location, sex, race, age, size, histologic grade, pT stage, pN stage, mpN stage, and TDs as significantly correlated with OS (log-rank tests, all *P* < 0.05). However, because mpN stage can be regarded as an adjusted categorization of pN stage, these stages were highly correlated, and subsequent multivariate analyses were performed including either pN or mpN stage. Both the pN and mpN stage were identified as independent prognostic factors for OS by multivariate analyses (all *P* < 0.001) (Tables 2, 3). Similar

**Table 2:** Multivariate analysis of overall survival in colorectal cancer patients with pN stage.

**Variables**	**Training cohort**		**Validation cohort**	
	**HR (95% CI)**	***P* value**	**HR (95% CI)**	***P* value**
Location	0.969 (0.877–1.071)	0.539	0.954 (0.820–1.111)	0.546
Sex	1.131 (1.049–1.219)	0.001	1.208 (1.075–1.357)	0.001
Race	0.998 (0.946–1.052)	0.938	0.975 (0.898–1.058)	0.546
Age	1.689 (1.565–1.822)	<0.001	1.607 (1.429–1.807)	<0.001
Size	1.112 (1.039–1.191)	0.002	1.152 (1.036–1.280)	0.009
Grade	1.227 (1.168–1.290)	<0.001	1.246 (1.157–1.343)	<0.001
T stage	1.538 (1.469–1.611)	<0.001	1.428 (1.333–1.531)	<0.001
TD status	1.031 (1.023–1.039)	<0.001	1.027 (1.014–1.039)	<0.001
pN stage	1.204 (1.175–1.235)	<0.001	1.225 (1.178–1.273)	<0.001

*CI, confidence interval; HR, hazard ratios; TD, tumor deposit*.

**Table 3:** Multivariate analysis of overall survival in colorectal cancer patients with mpN stage.

**Variables**	**Training cohort**		**Validation cohort**	
	**HR (95% CI)**	***P*-value**	**HR (95%CI)**	***P*-value**
Location	0.954 (0.863–1.054)	0.351	0.943 (0.810–1.098)	0.449
Sex	1.129 (1.048–1.217)	0.001	1.210 (1.077–1.360)	0.001
Race	0.999 (0.947–1.053)	0.961	0.977 (0.900–1.060)	0.569
Age	1.690 (1.566–1.823)	<0.001	1.618 (1.439–1.819)	<0.001
Size	1.112 (1.039–1.191)	0.002	1.159 (1.042–1.288)	0.006
Grade	1.224 (1.165–1.286)	<0.001	1.244 (1.155–1.341)	<0.001
T stage	1.524 (1.455–1.597)	<0.001	1.409 (1.314–1.511)	<0.001
TD status	1.025 (1.016–1.034)	<0.001	1.019 (1.006–1.033)	0.006
mpN stage	1.339 (1.292–1.388)	<0.001	1.393 (1.318–1.473)	<0.001

*CI, confidence interval; HR, hazard ratios; mpN, modified pN; mTNM, modified TNM; TD, tumor deposit*.

findings were observed in the validation cohort (Tables 2, 3).

### Upstaging After Applying TDs as pLNs

**Figure 2:**
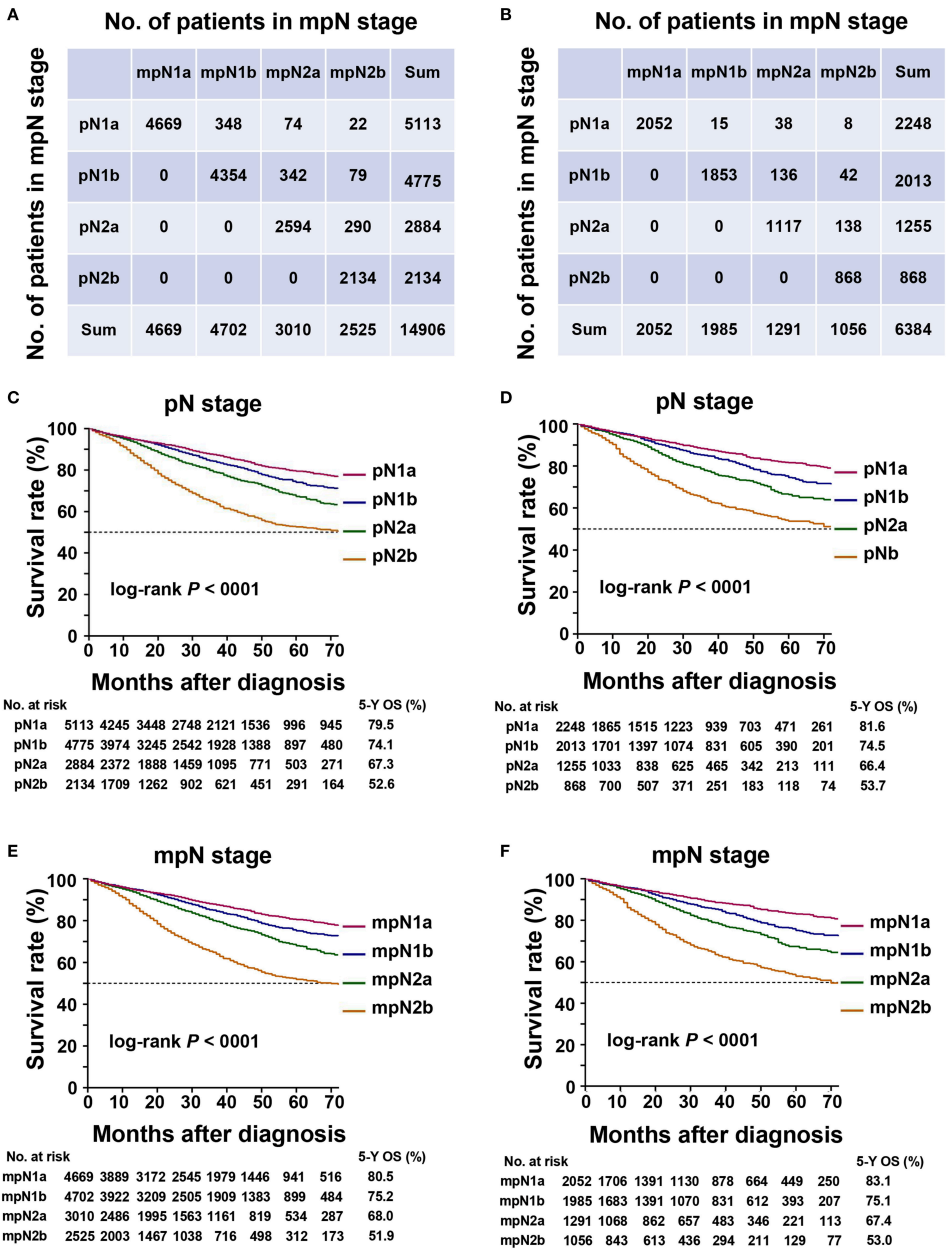
Staging migration and survival curves based on the pN stage and mpN stage. **(A)** staging migration between the tumor-node-metastases (TNM) and mTNM classification in the training cohort; **(B)** staging migration between the TNM and mTNM classification in the validation cohort; **(C)** survival curve for pN stage in the training cohort; **(D)** survival curve for mpN stage in the training cohort; **(E)** survival curve for pN staging in the validation cohort; **(F)** survival curve for mpN stage in the validation cohort.

The current study demonstrated that some patients experienced upstaging after applying TDs as number of pLNs. In the training cohort, 7.7% of patients experienced upstaging in the pN stage (Figure 2A), including 8.7% of patients in pN1a, 8.8% in pN1b, and 10.0% in pN2a stages.

In the validation cohort, 5.9% of the patients in the pN stage experienced upstaging (Figure 2B), including 2.7% of the patients in the pN1a, 8.8% in the pN1b, and 11.3% in the pN2a stages.

### OS According to pN and mpN Stages

In the training cohort, the 5-year OS of patients with stages pN1a, pN1b, pN2a, and pN2b were 79.5, 74.1, 67.3, and 52.6%, respectively (Table 4, Figure 2C) (log-rank test, overall *P* < 0.001). The 5-year OS rates of patients with stages mpN1a, mpN1b, mpN2a, and mpN2b were 80.5, 75.2, 68.0, and 51.9%, respectively (Table 4, Figure 2E) (log-rank test, overall *P* < 0.001). Similar findings were observed in the validation cohort (Table 4, Figures 2D,F) (log-rank *P* < 0.001).

**Table 4:** Three- and five-year OS and 95% CI for pN stage, mpN stage, TNM staging system, and mTNM staging system in training and validation cohorts.

**Variables**	**No. of patients (%)**	**HR (95% CI)**	**3-Y OS (%)**	**5-Y OS (%)**	***P* value**
**Training cohort**					
pN stage					<0.001
pN1a	5,113 (34.3)	1 (Reference)	87.5	79.5	
pN1b	4,775 (32.0)	1.247 (1.126–1.381)	84.2	74.1	
pN2a	2,884 (19.3)	1.677 (1.504–1.870)	79.1	67.3	
pN2b	2,134 (14.3)	2.891 (2.601–3.213)	64.4	52.6	
mpN stage					<0.001
mpN1a	4,669 (31.3)	1 (Reference)	87.9	80.5	
mpN1b	4,702 (31.5)	1.234 (1.108–1.374)	85.0	75.2	
mpN2a	3,010 (20.2)	1.689 (1.511–1.889)	80.0	68.0	
mpN2b	2,525 (16.9)	3.064 (2.758–3.405)	64.8	51.9	
TNM staging system					<0.001
IIIA	2,277 (15.3)	1 (Reference)	92.9	87.4	
IIIB	9,473 (63.6)	2.143 (1.842–2.493)	84.4	74.1	
IIIC	3,156 (21.2)	4.989 (4.270–5.829)	64.5	52.2	
mTNM staging system					<0.001
IIIA	2,247 (15.1)	1 (Reference)	92.9	87.7	
IIIB	9,144 (61.3)	2.099 (1.799–2.448)	85.0	74.9	
IIIC	3,515 (23.6)	5.117 (4.375–5.986)	64.9	51.7	
**Validation cohort**					
pN stage					<0.001
pN1a	2,248 (35.2)	1 (Reference)	88.2	81.6	
pN1b	2,013 (31.5)	1.348 (1.149–1.582)	85.1	74.5	
pN2a	1,255 (19.7)	1.878 (1.588–2.220)	77.9	66.4	
pN2b	868 (13.6)	3.184 (2.698–3.758)	64.5	53.7	
mpN stage					<0.001
mpN1a	2,052 (32.1)	1 (Reference)	89.0	83.1	
mpN1b	1,985 (31.1)	1.426 (1.205–1.688)	85.6	75.1	
mpN2a	1,291 (20.2)	1.956 (1.641–2.331)	79.0	67.4	
mpN2b	1,056 (16.5)	3.516 (2.977–4.153)	64.7	53.0	
TNM staging system					<0.001
IIIA	983 (15.4)	1 (Reference)	92.2	87.4	
IIIB	4,073 (63.8)	2.02 (1.611–2.533)	84.8	74.7	
IIIC	1,328 (20.8)	4.608 (3.650–5.818)	65.6	54.6	
mTNM staging system					<0.001
IIIA	964 (15.1)	1 (Reference)	92.2	88.0	
IIIB	3,929 (61.5)	1.994 (1.581–2.514)	85.5	75.6	
IIIC	1,491 (23.4)	4.805 (3.795–6.085)	65.6	53.9	

*CI, confidence interval; HR, hazard ratios; mpN, modified pN; mTNM, modified TNM; No., number; OS, overall survival; 5-Y, 5-year*.

### OS According to AJCC TNM and mTNM Classifications

**Figure 3:**
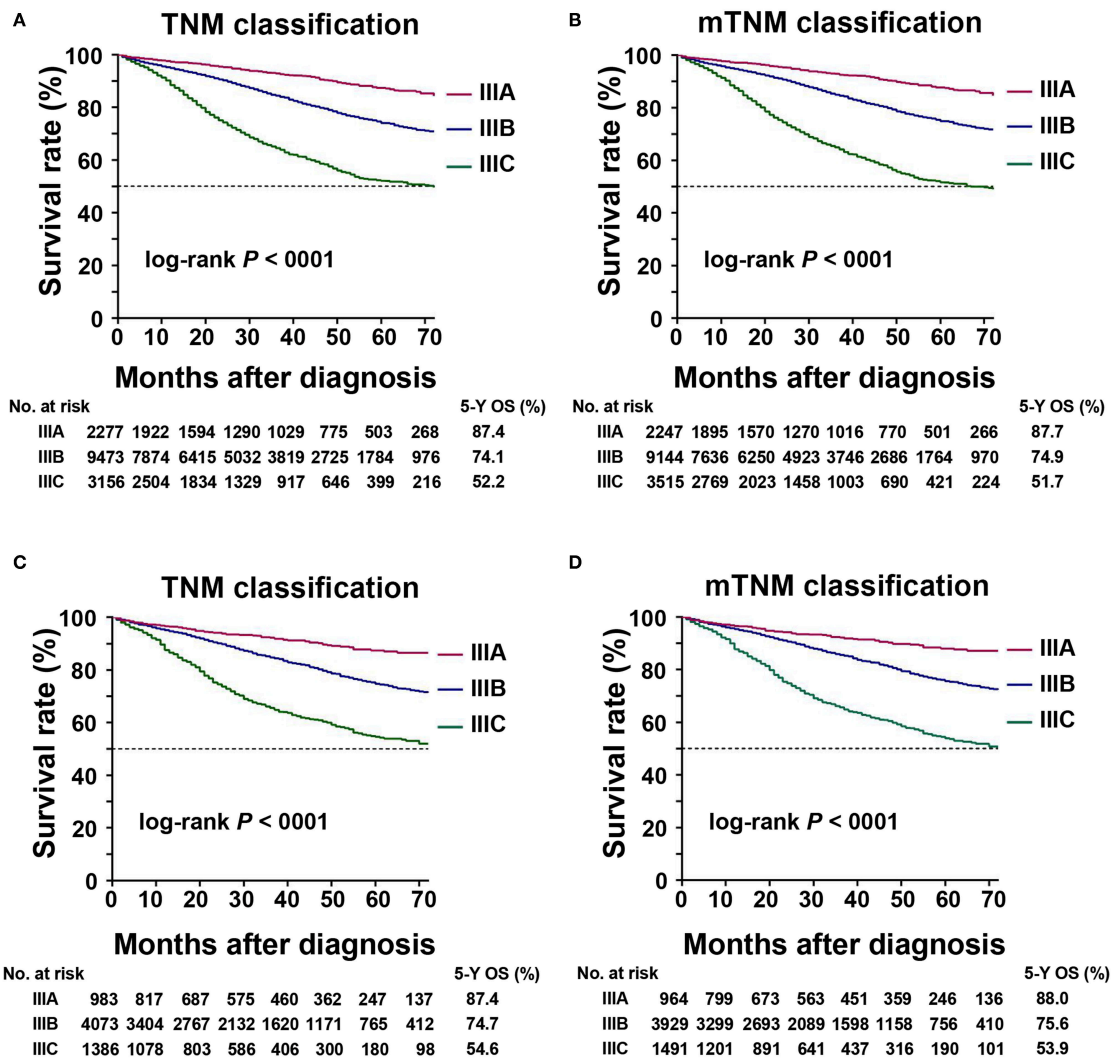
Kaplan–Meier survival curve for 5-year overall survival (OS) based on the TNM and mTNM classifications. **(A)** Kaplan–Meier survival curves based on TNM classification in training cohort; **(B)** Kaplan–Meier survival curves based on the mTNM classification in the training cohort; **(C)** Kaplan–Meier survival curves based on the TNM classification in the validation cohort; **(D)** Kaplan–Meier survival curves based on the mTNM classification in the validation cohort.

In the training cohort, the 5-year OS in patients with AJCC TNM classification stages IIIA, IIIB, and IIIC were 87.4, 74.1, and 52.2%, respectively. According to the mTNM classification, the equivalent 5-year OS rates for stages IIIA, IIIB, and IIIC were 87.7, 74.9, and 51.7%, respectively. The differences between the AJCC TNM and mTNM classifications were significant (log-rank test, overall *P* < 0.001) (Table 4, Figures 3A,B). Similar findings were observed in the validation cohort (log-rank test, overall *P* < 0.001) (Table 4, Figures 3C,D).

### Weights of TDs and pLNs in Predicting OS

**Figure 4:**
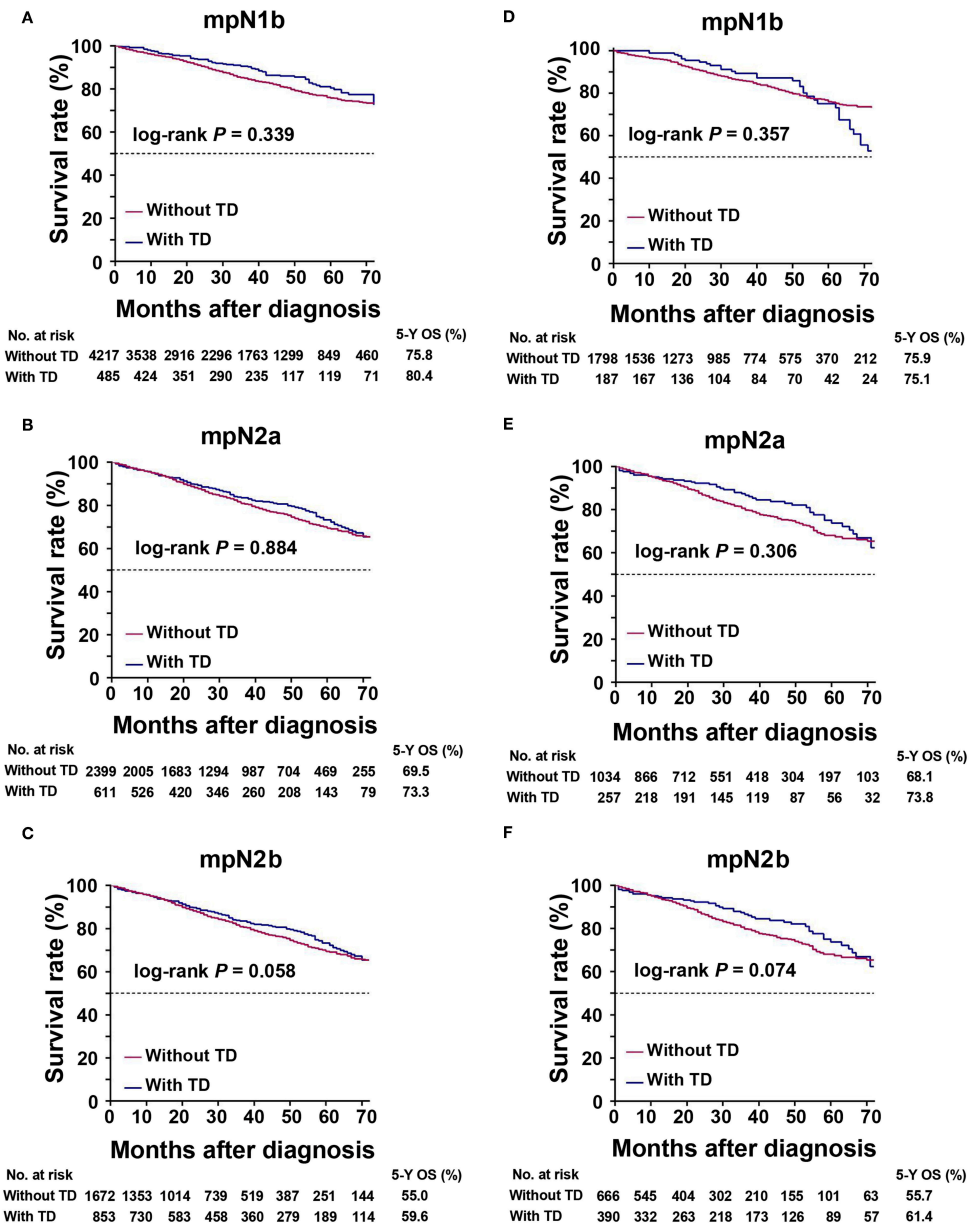
Kaplan–Meier survival curves for patients with or without tumor deposits (TDs) in the same mpN stages. **(A)** mpN1b in the training cohort; **(B)** mpN2a in the training cohort; **(C)** mpN2b in the training cohort; **(D)** mpN1b in the validation cohort; **(E)** mpN2a in the validation cohort; **(F)** mpN2b in the validation cohort.

To determine if TDs and pLNs had similar weights for predicting patient prognosis, we compared the OS of patients with pure pLNs and those with pLNs plus TDs (Figure 4). There was no prognostic heterogeneity, indicating that TDs had the same weight as pLNs (log-rank test, all *P* > 0.05).

### Comparison of Prognostic Performance Between pN and mpN Stages

**Figure 5:**
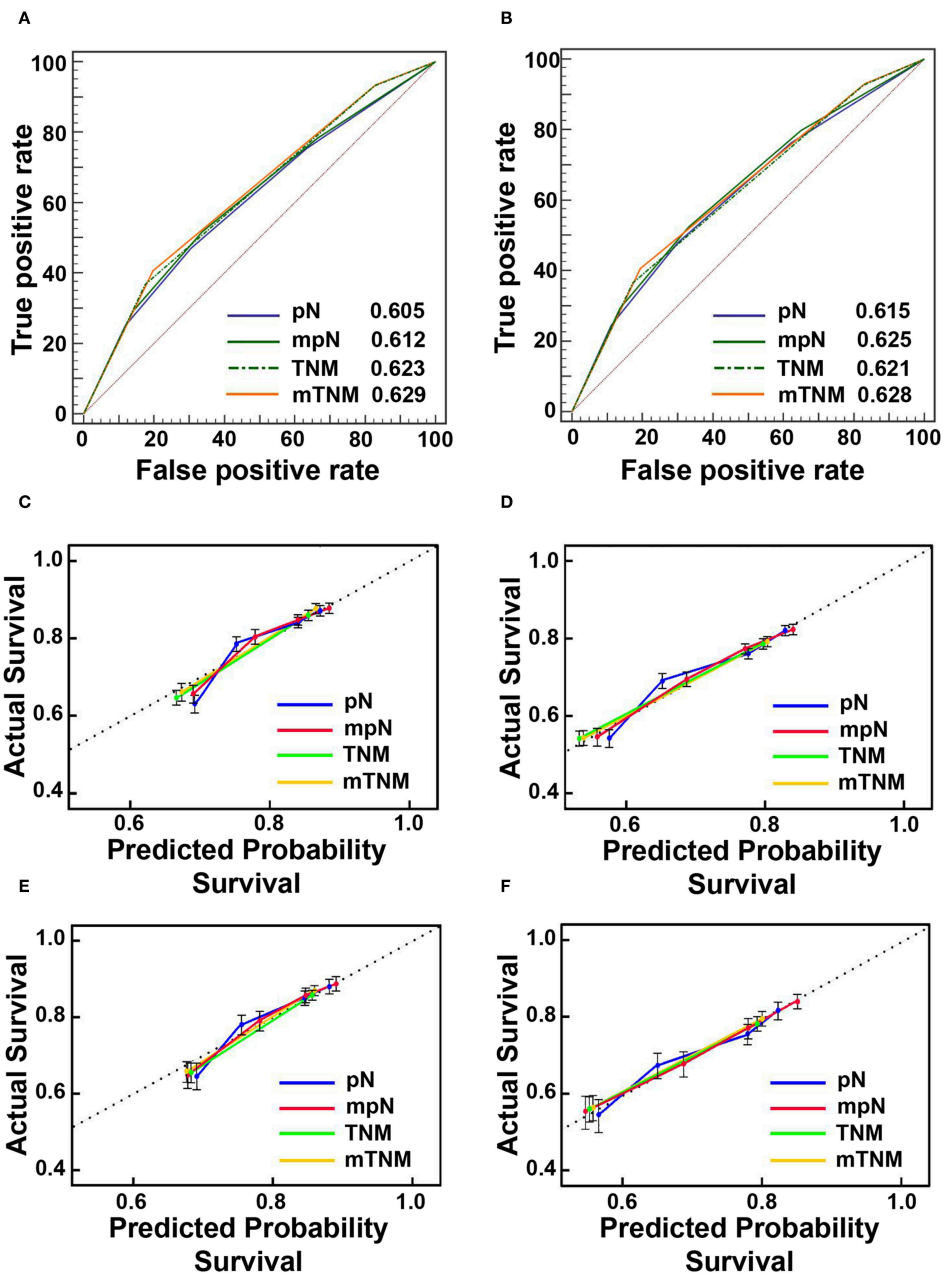
The areas under the curves (AUCs) and calibration curves for predicting patient survival. **(A)** AUCs in the training cohort; **(B)** AUCs in the validation cohort; **(C)** calibration curves of 3-year overall survival (OS) in the training cohort; **(D)** Calibration curves of 5-year OS in the training cohort; **(E)** Calibration curves of 3-year OS in the validation cohort; **(F)** calibration curves of 5-year OS in the validation cohort.

In the training cohort, the mpN stage showed superior prognostic discrimination [AUC 0.612, 95% confidence interval (CI), 0.604–0.620] compared with the pN stage (AUC 0.605, 95% CI, 0.597–0.612) (Hanley and McNeil test, *P* < 0.001, Figure 5A), and the mpN stage also showed better model fitting than the pN stage (AIC, 49,756 vs. 49,841). The calibration curves for probability of survival in the mpN stage at 3 and 5 years also showed better consistency between the predicted and observed survival than the pN stage (Figures 5C,D). Similar results were found in the validation cohort (Table 5, Figures 5B,E,F).

**Table 5:** Prognostic performances of pN stage, mpN stage, TNM staging system, and mTNM staging system in training and validation cohorts.

**Variables**	**Area under the curve (AUC) (95% CI%)**	**Akaike's information criterion (AIC)**	*P-*value^*^
**Training cohort**			
pN stage	0.605 (0.597–0.612)	49,841	
mpN stage	0.612 (0.604–0.620)	49,756	<0.001
**Training cohort**			
TNM staging system	0.623 (0.615–0.631)	49,563	
mTNM staging system	0.629 (0.621–0.637)	49,495	0.001
**Validation cohort**			
pN stage	0.615 (0.603–0.627)	18,843	
mpN stage	0.625 (0.613–0.637)	18,795	0.004
**Validation cohort**			
TNM staging system	0.621 (0.609–0.633)	18,773	
mTNM staging system	0.628 (0.616–0.639)	18,740	0.024

*AUC, Areas under the receiver-operating characteristic curve; AIC, Akaike's information criterion; CI, confidence interval; mpN, modified pN; mTNM, modified TNM*.

*A higher AUC indicates better discrimination and a lower AIC indicates superior model-fitting*.

* *P-value of Hanley & McNeil test*.

### Comparison of Prognostic Performance Between TNM and mTNM Classifications

In the training cohort, the mTNM classification showed superior prognostic discrimination (AUC 0.629, 95% CI, 0.621–0.637) compared with the TNM stage (AUC 0.623, 95% CI, 0.615–0.631) (Hanley and McNeil test, *P* = 0.001, Figure 5A), and the mTNM stage showed better model fitting than the TNM stage (AIC, 49,495 vs. 49,563). The calibration curves for probability of survival in the mTNM classification at 3 and 5 years also showed better consistency between the predicted and observed survival than the AJCC TNM classification (Figures 5C,D). Similar results were found in the validation cohort (Table 5, Figures 5B,E,F).

### Clinical Use

**Figure 6:**
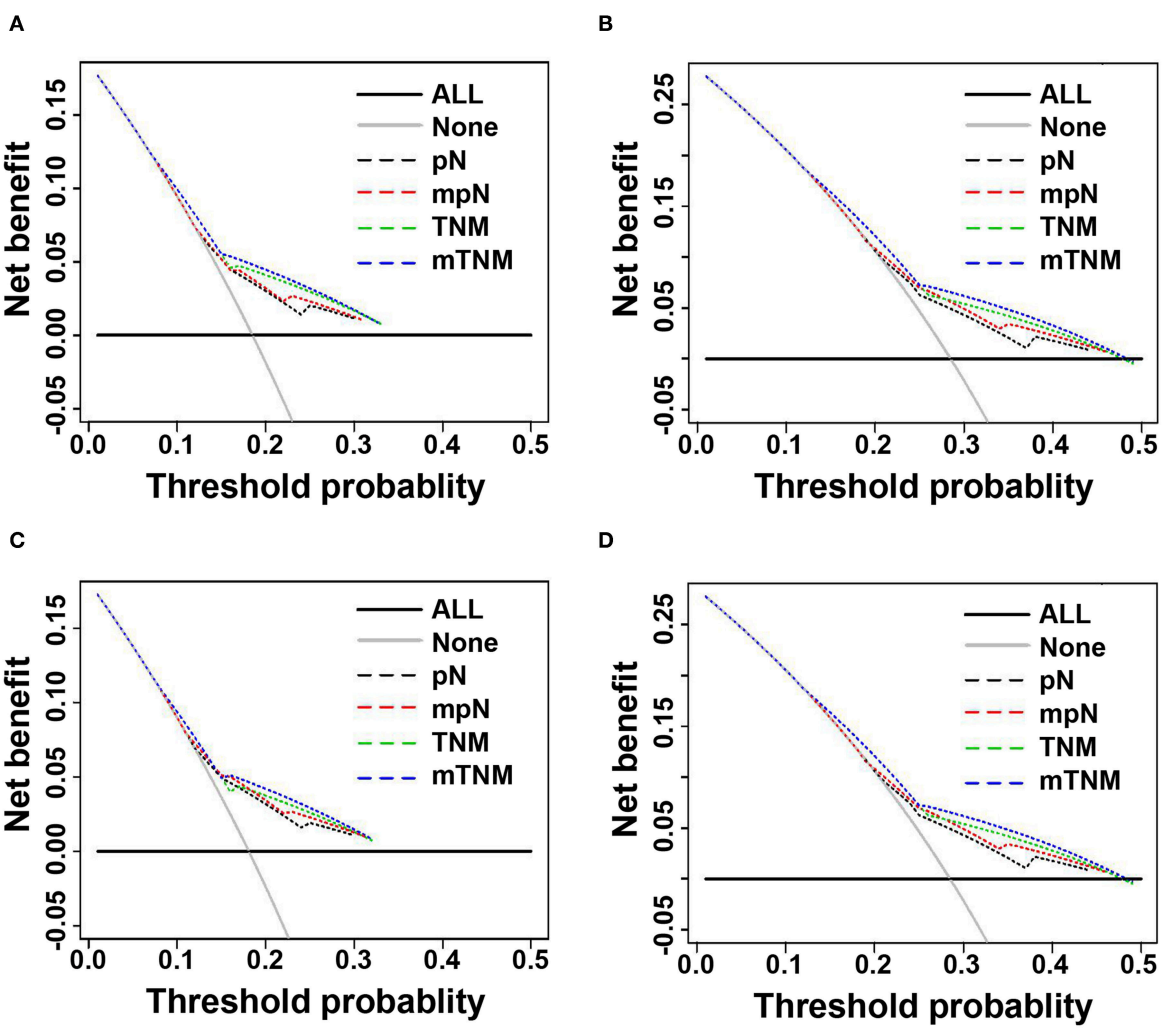
Decision curve analysis (DCA) of 3- and 5-years overall survival (OS) of pN phase, mpN stages, TNM classification, and mTNM classification. **(A)** DCAs of 3-year OS in the training cohort; **(B)** DCAs of 5-year OS in the training cohort; **(C)** DCAs of 3-year OS in the validation cohort; **(D)** DCAs of 5-year OS in the validation cohort.

We used DCAs to evaluate the clinical usefulness of the pN stage, the mpN stage, the TNM classification, and the mTNM classification in the training and validation cohorts. In the training and validation cohorts, the mpN stage showed higher net benefit than the pN stage between threshold probabilities of about 15–25% in predicting 3-year OS, and between threshold probabilities of about 20–45% in predicting 5-year OS (Figure 6). In the training and validation cohorts, the mTNM classification showed higher net benefit than the AJCC TNM classification between threshold probabilities of about 15–30% in predicting 3-year OS, and between threshold probabilities of about 25–50% in predicting 5-year OS (Figure 6).

## Discussion

The prognosis of patients with CRC has gradually improved in recent decades, however, many issues remain to be resolved. Changes in the definition of TDs have caused considerable confusion among researchers and have had a significant impact on the choices of post-operative treatment and on precise prognosis (13). Gabriel et al. depicted TDs as a result of vascular tumor dissemination in 1935, however, given that TDs were located in the pericolic or perirectal fattiness not continuous with the primary tumor and unassociated with nodes, it was difficult to distinguish between TDs and LNs (18). The AJCC 5th TNM classification of CRC, therefore, proposed a 3-mm rule for positive TDs: TDs >3 mm were categorized as pLNs, and TDs ≤ 3 mm were categorized as discontinuous stage T3 tumor (3). However, the AJCC 6th TNM classification redefined TDs based on contour: the shape and glossy contour of TDs with histological proof of LNs were categorized as pLNs, while irregular TDs were categorized as pT classification and venous invasion (4). However, this contour-based rule remained unconvincing (19).

Currently, the AJCC 7th TNM classification suggests that T1 and T2 lesions without pLNs, but with TDs, are categorized as N1c, though this is not consistent because pN1c is also an option for pT3 or pT4a tumors in CRC staging (5). The AJCC 8th TNM for CRC kept the N1c stage unchanged (6) and did not provide a definition of TDs, with an impact on repeatability as a result of individual judgments by different pathologists. However, despite several changes in the TNM classification, it is still regarded as the most formidable and dependable predictor of prognosis for CRC patients worldwide (12). One study showed that the 7th TNM classification was a good predictor of prognosis in CRC patients without LN metastasis and with TDs (20). However, the latest TNM classification failed to provide further staging recommendations for patients with both TDs and pLNs, with implications for the accurate categorization of these patients.

The AJCC 8th TNM classification of gastric cancer regarded TDs as pLNs, and the number of TDs was included for pathological staging (6). Furthermore, TDs were regarded as pLNs in the Japanese categorization of CRC (21). Based on the above studies, we therefore regarded TDs as pLNs and re-identified the pN stage. Univariate analysis showed that the pN and mpN stages were both significantly related to prognosis among patients with CRC, while multivariate analysis identified both the pN and mpN stages as independent risk factors for the prognosis of these patients. The 5-year OS of patients with and without TDs were 54.8 and 73.5%, respectively (log-rank test, *P* < 0.001), indicating that patients with TDs had a poorer prognosis than those without TDs. Similar findings were obtained in the validation cohort and in previous studies (19, 22–24). Some patients experienced upstaging due to changes in the definition of the mpN stage. There was a clear tendency for CRC patients with TDs in the pN subgroups to be upstaged to higher stages in the mTNM classification. We determined if the mpN stage and mTNM classification were better than the pN stage and TNM classification in terms of predictive power by AUC and AIC analysis, which showed that the mpN stage and mTNM classification were more powerful than the pN stage and TNM classification in terms of prediction ability. The mpN stage and mTNM classification had a lower AIC and higher AUC (Hanley and McNeil test, all *P* < 0.001), indicating that in terms of predictive ability, they were more powerful than the other two models. We also analyzed the clinical benefits of the TNM and mTNM classifications by DCA, and showed that the mTNM classification had better clinical benefits than the TNM classification. In addition, there was no significant difference in the prognosis between CRC patients with and without TDs in the same mpN stage (log-rank test, all *P* > 0.05). The results further showed that TDs were of equal importance to pLNs in predicting the prognosis of patients with CRC. We therefore recommend regarding TDs as pLNs in the TNM classification, with the mpN stage having a stronger ability to predict the prognosis of CRC than the pN stage.

Opinions on the origin of TDs differ. One study suggested that TDs originated from carcinomas growing inside or along lymphatic or vascular structures or nerves (19), while another study indicated that TDs were potential pLNs, which were no more knew because they were replaced by tumor cells (7). Irrespective of their size, TDs were shown to have a significant impact on disease-free survival in patients with CRC, suggesting that TDs of all sizes should be regarded similarly (25). The current results showed that the mpN category and mTNM classification were superior to the pN category and TNM classification in assessing the prognosis and survival of CRC patients. In light of these results, TDs appear to have a negative effect on prognosis and similar effects on survival as pLNs.

Using the SEER database (14) makes it possible to draw reasonable conclusions consistent with general clinical practice based on a large sample of CRC patients, which would be impossible to realize in a single institutional study. However, this study had some limitations. First, even though the SEER database was regularly checked for differences, the possibility of coding or data errors remains. Second, although this study was based on a large database, it had limitations associated with its retrospective nature. In addition, the results may not be applicable in Asian populations because it was based on the SEER database of Western population, and there was no exact information of the exact number of Asian patients. Therefore, the current findings still require further validations in the Asian population. Besides, the demographic and pathological characteristics of CRC patients in Asian and Western countries are different; therefore, the current findings need to be cautious before applying it in clinical practice, particularly in Asian populations. Finally, although the difference between the two CRC classifications is statistically significant, the absolute values of AUCs are small. Therefore, the results of this study should be cautious before applying it in clinical practice and require further verification.

## Conclusions

In conclusion, the current findings suggest that TDs should be regarded as pLNs when assessing the prognosis of patients with CRC. The proposed mpN stage and mTNM classification may be superior to the AJCC 8th pN stage and TNM classification for evaluating the prognosis and survival of patients with CRC.

## Data Availability Statement

The raw data supporting the conclusions of this article will be made available by the authors, without undue reservation.

## Ethics Statement

A data use agreement with SEER has been obtained. The approval of institutional review board was not required as the SEER database holds publicly available de-identified data. Written informed consent for participation was not required for this study in accordance with the national legislation and the institutional requirements.

## Author Contributions

J-PP and C-DZ wrote the main text and performed data analysis. J-PP, C-DZ, and D-QD designed the study. J-PP, C-DZ, XF, YB, SY, and Z-MZ collected the data. All authors reviewed the manuscript.

## Conflict of Interest

The authors declare that the research was conducted in the absence of any commercial or financial relationships that could be construed as a potential conflict of interest.
